# Westernized and Diverse Dietary Patterns Are Associated With Overweight-Obesity and Abdominal Obesity in Mexican Adult Men

**DOI:** 10.3389/fnut.2022.891609

**Published:** 2022-06-24

**Authors:** Sonia Rodríguez-Ramírez, Brenda Martinez-Tapia, Dinorah González-Castell, Lucía Cuevas-Nasu, Teresa Shamah-Levy

**Affiliations:** ^1^Center for Research on Nutrition and Health, National Institute of Public Health, Cuernavaca, Mexico; ^2^Center for Research on Evaluation and Surveys, National Institute of Public Health, Cuernavaca, Mexico

**Keywords:** adult population, dietary patterns, cluster analysis, obesity, abdominal obesity, Mexico, gender difference

## Abstract

**Introduction:**

The prevalence of overweight and obesity in Mexican adults is very high. To identify the dietary characteristics related with this disorder is necessary to design intervention. The objective was to analyze the association between dietary patterns and obesity in Mexican adults.

**Materials and Methods:**

This is a cross-sectional study carried out in Mexican adults (20–59 years old) participating in the Halfway National Health and Nutrition Survey 2016. Participants (*n* = 5,735) were classified as having normal weight, overweight-obesity and by their abdominal circumference as having abdominal obesity or not. With information from a 7-day food frequency questionnaire, we used a K-means cluster analysis to derive dietary patterns and calculated a healthy diet indicator to evaluate quality. The association between dietary patterns and overweight-obesity and abdominal obesity was assessed with Poisson regression models adjusted by some characteristics.

**Results:**

We identified a Rural pattern characterized by tortilla, legumes and egg consumption; a Diverse pattern, characterized by fruits, meat and poultry, vegetables, and dairy beverages, and desserts; and a Westernized pattern, characterized by sweetened non-dairy beverages, fast food, bakery and cookies, candies and salty snacks. In men, Westernized pattern was associated with overweight-obesity (PR = 1.11, 95% CI 0.97–1.27), and abdominal obesity (PR = 1.15, 95% CI 1.00–1.33), the Diverse pattern was associated with overweight-obesity (PR = 1.18, 95% CI 1.00–1.38), and abdominal obesity (PR = 1.27, 95% CI 1.07–1.50), compared with the Rural pattern. In women, these dietary patterns were not associated with obesity.

**Discussion:**

Westernized and Diverse patterns are associated with overweight and obesity and abdominal obesity in men. Gender-specific recommendations and surveillance are necessary in the Mexican adult population.

## Introduction

According to national estimates, the prevalence of overweight and obesity has increased in Mexico over the past decades. The increase was more pronounced between 2000 and 2006, compared to the 2006–2012 period (1). In 2016, the prevalence in adult population rose to 72.5% (2) and reached 75.2% (3) in 2018.

This increase has been accompanied by non-communicable chronic diseases as diabetes and hypertension. For instance, in 2016, the prevalence of type 2 diabetes was 9.4% (previously diagnosed) (4) and 25.5% for hypertension. Of those with hypertension, 40% did not know they had the condition (5). Furthermore, there is evidence that increase of body mass index is associated with increased rates of type 2 diabetes, pre-diabetes, and hypertension in Mexican population (6).

The relationship between diet and obesity has been well-established in the literature. Studies on the dietary factors associated with excess weight and obesity conducted in the past decades focused on specific dietary components such as macronutrients and fiber (7). However, foods are consumed in complex combinations that can have synergistic or antagonistic effects and it is difficult to isolate the impact of individual foods and nutrients (8).

Overweight and obesity are caused by the interaction between genetics, environment, and human behavior. When energy intake exceeds energy expenditure, adipose tissue accumulates, and can eventually lead to obesity. Furthermore, the evidence show that dietary patterns characterized by high carbohydrate from refined grains are associated with obesity due to high glycemic index carbohydrates causes rapid changes in blood glucose and insulin levels, and sugar causes addictive cravings, glucose, and insulin signal the midbrain limbic system to change dopamine levels triggering inducement of food addiction (9).

To address these issues, several authors have proposed to study overall dietary patterns by considering how foods and nutrients are consumed together. The association between dietary patterns and obesity has been investigated in various studies (9–12), showing an association between dietary patterns characterized mainly by foods high in fat, meat, dairy, and processed foods and obesity. Likewise, certain food groups have been shown to have a protective effect against obesity such as fruits, vegetables, and fish (11).

Many studies have analyzed gender differences on the association between dietary patterns and disease risk, founding inconsistent results (13–15). In Mexico, there are few studies that have explored the relationship between dietary patterns and obesity by gender (16–19). For example, a study carried out in adult (20–59 years old) participants of the National Health and Nutrition Survey 2006, described three dietary patterns and found that individuals who consumed what they called a traditional dietary pattern based on maize and maize foods, beans and legumes, had lower Body Mass Index (BMI), and higher physical activity than the other two patterns (19).

Due to the sustained increase in the prevalence of obesity in Mexico, it becomes relevant to identify the characteristics of the dietary pattern that increase the possibility to present obesity in the different genders. Therefore, the objective of this study was to analyze the association of dietary patterns and overweight and obesity and abdominal obesity in Mexican adult (20–59 years old) participants from the Halfway National Health and Nutrition Survey 2016 (ENSANUT MC-2016 by its acronym in Spanish).

## Materials and Methods

### Study Population

This is a cross-sectional study from the ENSANUT MC-2016, which is a probabilistic, multistage, stratified survey that has national, regional and urban/rural area representativeness. Data collection was performed from May to October 2016. A detailed description of the design and sampling procedures has been published by Romero-Martínez et al. (20).

### Dietary Information

The dietary data was obtained from a random subsample of the adult population aged 20–59 years old (*n* = 6,196).

Trained personnel administered a 7-day food frequency semi quantitative questionnaire (FFQ), which included information from 140 foods and beverages. This questionnaire was previously validated for energy and nutrient intake in adult population (21). Through days, times per day, portion size, and number of portions consumed, consumption was estimated in grams. Data of consumption > 4 standard deviations (SD) above the mean by gender, area and region for each food or beverage were considered implausible and the mean consumption was imputed (22). Participants with ≥7 foods and beverages with imputed consumption were excluded from the analysis (*n* = 45). Afterwards, energy intake was estimated through a nutrient database compiled by the INSP [National Public Health Institute 2013, Database on food nutritional value, compiled by Instituto Nacional de Salud Publica (Unpublished)^1^].

Energy requirement predicted (pER) was estimated through the Institute of Medicine equations (23) and the Mifflin-St Jeor equation was used to estimate resting metabolic rate (24). Participants who had a ratio of intake/energy requirement (EI/pER) >3 SD above the mean by sex were excluded (*n* = 54), as well as those with energy intake under half of their resting metabolic rate (*n* = 165).

We classified food and beverages in 25 groups according to their nutritional characteristics and cooking procedures, excluding plain water: (1) Dairy sweetened beverages, (2) Dairy non-sweetened beverages, (3) Sweetened non-dairy beverages, (4) Non-sweetened, non-dairy beverages, (5) Fruits, (6) Vegetables, (7) Non-beverage dairy products, (8) Legumes, (9) Cereal based salty dishes, (10) Corn based salty dishes, (11) Fast food, (12) Egg, (13) Meat and poultry, (14) Processed meat, (15) Bakery and cookies, (16) Candies, (17) Dessert, (18) Salty snacks, (19) Seeds, (20) Added fats, (21) Tortilla, (22) Soups, (23) Ready to eat cereals, (24) Bread, and (25) Potatoes (Example of the food included in each group are in Supplementary Table 1).

### Dietary Patterns

The energy contribution (%) by each food group was estimated and a standardized transformation from the contribution variables was made by subtracting the mean and dividing by the SD. This variable was used in a cluster analysis, by k-means to classify adults in non-overlapping groups, based on their eating patterns. We tested 2–6 solutions to select the number of clusters for analysis by comparing the Calinski-Harabasz index (CH index). The solution with the highest CH index is considered the most optimal solution based on the average between- and within-cluster sum of squares (25). In addition, nutritional interpretability and sample size were also considered.

### Healthy Diet Indicator (HDI)

This index is based on the World Health Organization chronic disease prevention diet and nutrition guides (26, 27). It contains nine components, with dichotomous answers according to the compliance of each one (0 = not compliant; 1 = compliant); the maximum score was 9 points (28). These components and adherence values (score = 1) were: saturated fat (≤ 10% of energy), polyunsaturated fat (6–10% of energy), protein (10–15% of energy) and carbohydrates (≥50– ≤ 70% of energy), fiber (>25 g), fruits and vegetables (>400 g), consumption of legumes and seeds (>30 g), and cholesterol intake (≤ 300 mg), and sugar (≤ 10% of energy). The free sugars were not available in this database and were replaced by mono- and disaccharides, as suggested in another study (28). We estimated the percentage of participants who met each component of the HDI, the score as a continuous variable for each participant and estimated the mean of the HDI score into each dietary pattern.

### Anthropometric Information

Personnel were trained according to international procedures to measure height, weight and waist circumference (29, 30). Bodyweight was measured using an electronic scale Seca model-874 (200 kg and a precision of 100 g, Hamburg, Germany) and the height using a stadiometer Seca model-206 (220 cm and a precision of 1 mm Hamburg, Germany).

We excluded 103 participants without anthropometric information. Valid height values were considered between 1.3 and 2.0 m and the BMI values between 10 and 58 kg/m^2^. Data beyond these intervals were excluded from the analysis (n = 94).

Regarding waist circumference, valid values were considered between 50 and 200 cm. Data outside of this interval were excluded (*n* = 34).

**Figure 1:**
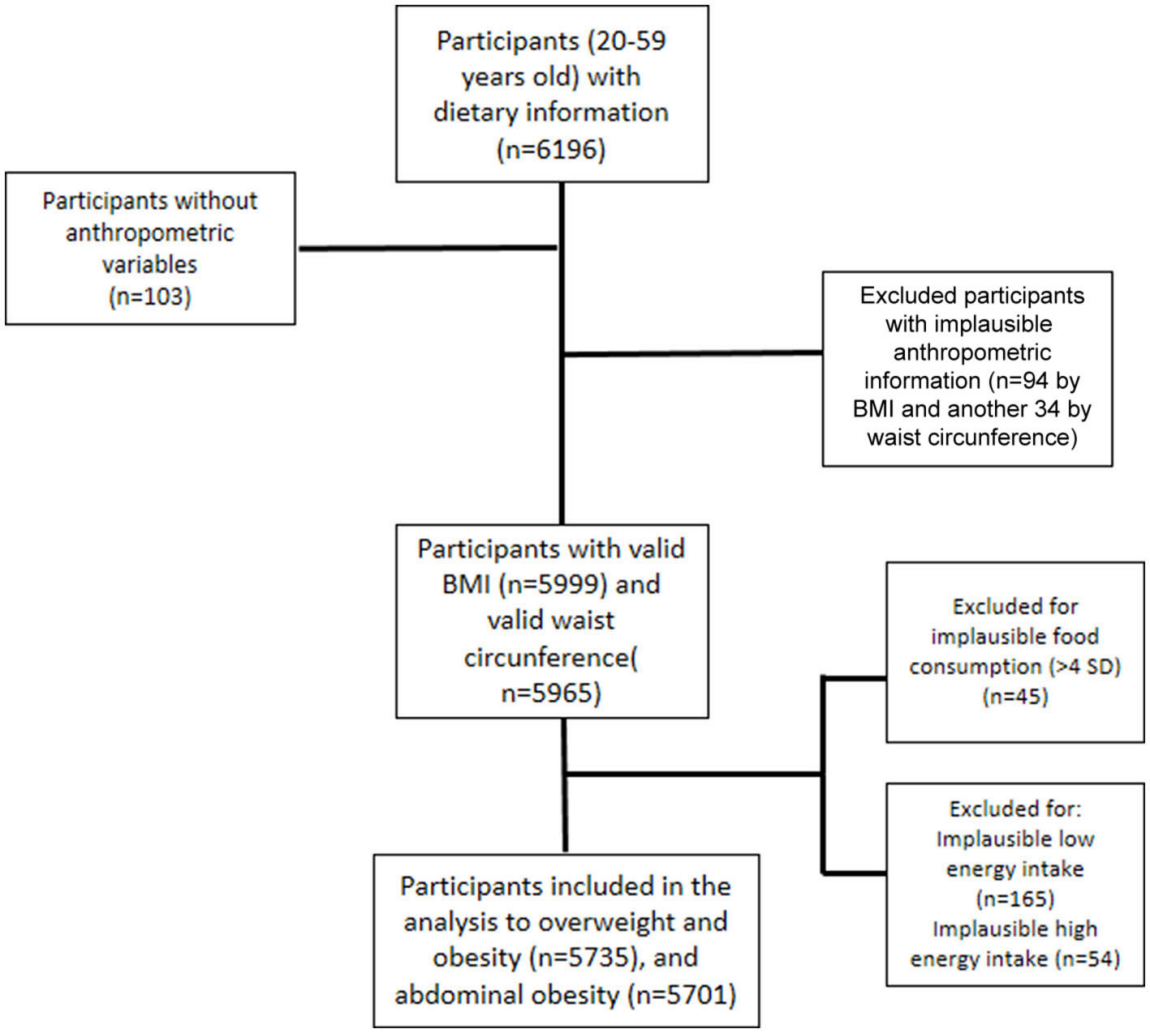
Flow chart of sample size of Mexican adults, National Health and Nutrition Survey 2016.

The final sample of study with valid anthropometric information and valid dietary intake was 5,735 adults (Figure 1).

BMI was categorized according to WHO cut-off points (normal weight if BMI <25 and overweight and obesity if BMI ≥ 25 kg/m^2^) (31) and abdominal obesity was classified according to the International Diabetes Federation (≥80 and ≥90 centimeters in women and men, respectively) (32).

### Sociodemographic Variables

Age was calculated in years at the date of the interview.

#### Geographical Region

The 32 states of the country were divided in four regions: North, Center, Mexico City, and South, using the previous classification used by the ENSANUT (20).

#### Area

Localities with <2,500 inhabitants were considered rural areas and those with ≥2,500 inhabitants were urban.

#### Socioeconomic Status (SES)

It was estimated through principal component analysis with dwelling characteristics (roof, wall and floor materials, drainage, and water availability) and possessions like a television, computer, radio, phone, cable, refrigerator, microwave oven, stove, boiler, and washing machine. We obtained a continuous variable, which was divided into tertiles: low, medium, and high socioeconomic level (33). This methodology has been used in other ENSANUTs and is useful for considering inequality between participant households (34).

### Physical Activity

The international Physical Activity Questionnaire (IPAQ) short form was used to measure physical activity. This questionnaire included a period of 7 days, and has been previously validated in Mexican adults (35). Self-reported minutes per day were included for: (1) vigorous physical activity (VPA), (2) moderate physical activity (MPA), and (3) walking. Three categories of physical activity were included in the analysis: Inactive (participants with <150 min/w of MPA–VPA); moderate active (participants with 150–299 min/w of MPA–VPA; and very active (participants with ≥300 min/w of MPA–VPA.

### Ethics

The survey protocol was approved by the ethics board from the INSP and prior informed consent was obtained from all participants.

### Statistical Analysis

To analyze difference in characteristic of the population groups, we used *T*-test for continuous variables with normal distribution and chi square for categorical variables. To analyze differences of energy and nutrient intake between dietary patterns (which did not have a normal distribution), quantile regression models were used.

To analyze the association between dietary pattern (independent variables) and overweight-obesity and abdominal obesity (dependent variables), we used Poisson regression models adjusted by age, area, and region. Also, we included an interaction term of dietary pattern and gender. As a sensitivity analysis, we did the association analysis in the sample with physical activity information (*n* = 5,593).

For statistical difference by nutrition status and gender, the *p*-value considered was < 0.05. For multiple comparison among dietary patterns in dietary characteristics, the *p* < 0.016 was considered as significant (Bonferroni method) (36). In the models, *p* < 0.10 for the interaction term, was considered. All statistical analyses were done in STATA 14.0 (StataCorp, 2011. College Station, TX: Stata Press), considering the sample design of the survey.

## Results

We analyzed data from 5,735 participants, who represented 49,575,450 adults between 20 and 59 years old in Mexico. The 47% of the population was men (the 39% was men in the population with abdominal obesity). Three quarters of the population lived in urban area and 47% had high SES (Table 1).

**Table 1:** General characteristics of Mexican adults population National Health and Nutrition Survey 2016^†^.

**Characteristics**	**National^**‡**^**	**With normal weight^**§**^**	**With Overweight or obesity^**||**^**	**Without abdominal obesity^**¶**^**	**With abdominal obesity^**†**^^**†**^**
	**Mean (SE)**	**Mean (SE)**	**Mean (SE)**	**Mean (SE)**	**Mean (SE)**
Age (y)	37.1 ± 0.27	34.0 ± 0.50	38.3 ± 0.32*	32.9 ± 0.50	38.4 ± 0.31*
BMI (Kg/m^2^)	28.3 ± 0.13	22.4 ± 0.08	30.5 ± 0.13*	23.0 ± 0.12	30.0 ± 0.13*
Waist circumference (cm)^§§^	94.1 ± 0.32	81.3 ± 0.31	98.9 ± 0.33*	80.2 ± 0.29	98.6 ± 0.31*
	**% (CI 95%)**	**% (CI 95%)**	**% (CI 95%)**	**% (CI 95%)**	**% (CI 95%)**
Male sex	46.8 (44.4–49.0)	51.5 (47.3–55.8)	44.9 (42.2–47.7)	70.8 (66.7–74.7)	39.0 (36.4–41.7)*
Urban area	74.6 (71.6–77.4)	74.7 (70.9–78.1)	74.6 (71.4–77.6)	71.3 (66.9–75.3)	75.7 (72.5–78.7)
**Region**					
North	20.1 (17.4–23.2)	19.9 (16.2–24.2)	20.2 (17.4–23.4)	17.6 (13.4–22.8)	21.1 (18.1–24.4)
Center	33.8 (30.8–36.9)	37.9 (33.1–42.9)	32.2 (29.1–35.5)	34.0 (29.5–38.9)	33.5 (30.2–37.0)
Mexico City	15.4 (13.2–18.0)	14.0 (10.5–18.3)	16.0 (13.7–18.6)	15.6 (11.7–20.5)	15.4 (13.0–18.0)
South	30.6 (27.1–34.5)	28.2 (23.9–32.9)	31.6 (27.6–35.8)	32.8 (28.2–37.7)	30.0 (26.1–34.3)
**SES, %**					
Low	22.3 (19.9–25.0)	25.4 (21.5–29.7)	21.1 (18.5–24.0)	27.6 (23.5–32.1)	20.6 (18.1–23.3)*
Middle	30.6 (28.3–33.0)	28.2 (24.6–32.0)	31.5 (28.8–34.3)	30.9 (26.7–35.4)	30.4 (28.0–33.1)
High	47.1 (44.0–50.3)	46.4 (41.6–51.2)	45.6 (41.3–49.9)	41.5 (36.6–46.5)	48.9 (45.6–52.3)
**Physical activity** ^**|||**^					
Inactive	13.2 (11.6–14.9)	12.3 (9.7–15.4)	13.5 (11.7–15.5)	10.4 (7.9–13.6)	14.1 (12.1–16.2)
Moderate	9.5 (8.4–10.8)	9.7 (7.5–12.4)	9.5 (8.1–11.0)	9.6 (6.9–13.1)	9.5 (8.3–11.0)
Active	77.3 (75.1–79.3)	78.0 (74.3–81.3)	77.0 (74.6–79.2)	80.0 (75.9–83.6)	76.4 (73.9–78.7)

*BMI, Body mass index; WC, Waist circumference; SES, Socio-Economic status*.

*†Adjusted by sampling design*.

*^‡^Expanded sample = 49,575,450 adults*.

*^§^Expanded sample = 13,589,962 adults*.

*^||^Expanded sample = 35,985,488 adults*.

*^¶^Expanded sample = 12,076,628 adults*.

*Expanded sample = 37,316,040 adults*.

*^§§^Waist circumference measurement not available in 34 participants*.

*^||||^Physical activity measurement not available in 142 participants*.

*Significant difference compared with population without overweight or obesity, or abdominal obesity (P < 0.05).

We found three dietary patterns: Rural, Diverse, and Westernized. Compared with the other dietary patterns, the Rural pattern was characterized by higher energy intake percentage from tortillas (42.9%), legumes (4.5%), and eggs (4.0%); the Westernized pattern by sweetened non-dairy beverages (19.3%), fast food (5.7%), bakery and cookies (7.9%), bread (5.5%), and to a lesser extent, candies and salty snack groups (2.8%); and the Diverse pattern was characterized by a higher percentage of energy intake from fruits (9.5%), meat and poultry (8.9%), vegetables (5.9%), and dairy beverages (sweetened or not) (7.9%) (Table 2).

**Table 2:** Contribution of energy (%) by food group and dietary pattern.

	**Dietary pattern**					
	**Rural (%** **=** 26.9)*		**Westernized (%** **=** **42.5)**^**†**^		**Diverse (%** **=** **30.6)**^**‡**^	
**Food groups**	**Mean**	**SE**	**Mean**	**SE**	**Mean**	**SE**
Dairy sweetened beverages	1.39	0.07	1.84	0.08	3.27	0.14
Dairy non sweetened beverages	1.66	0.07	1.97	0.07	4.66	0.15
Sweetened non-dairy beverages	10.35	0.18	19.36	0.28	7.76	0.16
Non-sweetened, non-dairy beverages	0.87	0.04	0.84	0.04	1.6	0.06
Fruits	4.03	0.09	3.61	0.08	9.53	0.17
Vegetables	2.38	0.05	2.38	0.05	5.92	0.13
Dairy products no beverages	1.89	0.06	2.51	0.07	4.96	0.13
Legumes	4.55	0.09	1.97	0.05	2.69	0.06
Cereal based salty dishes	2.78	0.06	2.13	0.05	3.31	0.08
Corn based salty dishes	6.56	0.09	7.67	0.21	4.81	0.15
Fast food	1.02	0.05	5.66	0.14	3.18	0.11
Egg	4.05	0.1	3.76	0.09	3.54	0.09
Meat and poultry	3.76	3.76	6.42	0.12	8.89	0.15
Processed meat	1.05	0.04	1.70	0.05	1.47	0.05
Bakery and cookies	4.17	0.11	7.97	0.19	4.88	0.13
Candies	0.34	0.02	1.48	0.07	0.67	0.03
Desserts	0.62	0.03	1.4	0.06	1.97	0.08
Salty snacks	0.53	0.03	1.8	0.07	0.68	0.04
Seeds	0.27	0.02	0.63	0.04	0.53	0.04
Added fats	0.63	0.04	1.12	0.06	0.96	0.05
Tortilla	42.86	0.3	16.32	0.21	16.94	0.24
Soup	0.59	0.2	0.65	0.02	1.48	0.04
Ready to eat cereals	0.14	0.01	0.38	0.02	1.23	0.06
Bread	2.45	0.09	5.50	0.19	3.77	0.13
Potatoes	1.06	0.02	0.92	0.04	1.25	0.05

*Expanded population = 15,182,063 adults. †Expanded population = 21,064,950 adults. ^‡^Expanded population = 13,328,437 adults.

With respect to the contribution of energy and nutrients, men had a higher energy intake than women (2,206–2,644 kcal/d in men vs. 1,515–1,937 kcal/d in women among the three patterns, *P* < 0.05). The population with a Westernized pattern presented higher energy, carbohydrates, fat (total and saturated), sugar, and cholesterol intake compared with Rural and Diverse patterns (*p* < 0.05). Also, the population with a Westernized pattern showed lower fiber intake compared with the other dietary patterns (fiber intake in men was 26.9 g/d in Westernized Pattern, vs. 35.2 in Rural and 31.6 g/d in Diverse pattern; in women, it was 19.1 g/d in Westernized vs. 26.2 in Rural and 23.3 g/d in Diverse pattern, *p* < 0.016; Table 3).

**Table 3:** Daily energy and nutriments intake by dietary pattern and gender in Mexican adults (*n* = 5,735)^1^.

**Men**	**Dietary patterns**					
	**Rural**		**Westernized**		**Diverse**	
	**Median**	**p25, p75**	**Median**	**p25, p75**	**Median**	**p25, p75**
Energy (kcal)	2,206^a^	1,632, 2,709	2,644^b^	2,050, 3,487	2,242^a^	1,664, 3,024
Carbohydrates (g)	354.1^a^	275.1, 447.4	367.8^a^	286.3, 495.4	301.1^b^	231.4, 391.3
Fat (g)	51.8^a^	35.9, 70.9	79.9^b^	59.2, 110.3	74.7^b^	50.8,100.2
Fat (energy %)	21.8^a^	17.6, 25.4	29.6^b^	24.3, 33.4	30.0^b^	26.0, 34.8
Protein (g)	67.0^a^	48.8, 82.6	79.2^b^	60.6, 107.3	81.0^b^	(61.3, 109.3
Fiber (g)	35.2^a^	27.2, 47.5	26.9^b^	20.0, 36.3	31.6^a^	23.8, 41.3
Sugar (g)	86.9^a^	54.9, 113.5	155.5^b^	107.2, 217.9	115.2^c^	84.2, 173.7
Saturated fat (g)	16.2^a^	10.7, 23.4	29.4^b^	20.9, 38.7	27.7^b^	17.9, 37.4
Saturated fat (energy %)	6.7^a^	5.2, 9.0	10.3^b^	8.4, 12.0	10.7^b^	8.9, 13.3
Polyinsatured fat (g)	15.0^a^	10.2, 19.9	18.5^b^	13.4, 27.2	14.7^a^	10.1, 24.1
Polyinsatured fat (energy %)	6.0^a^	5.0, 7.6	6.7^b^	5.2, 8.9	6.1^a^	5.0, 7.5
Cholesterol (mg)	191.9^a^	99.5, 300.0	335.2^b^	207.6, 489.2	295.2^b^	177.1, 412.3
**Women**						
Energy (kcal)	1,515^a^	1,151, 1973	1,937^b^	1,448, 2,447	1,590^a^	1,174, 2,010
Carbohydrates (g)	246.3^a^	186.3, 314.9	263.8^a^	207.2, 350.7	224.5^b^	168.8, 285.9
Fat (g)	38.1^a^	27.4, 56.3	64.6^b^	46.5, 84.3	51.2^b^	36.1, 70.5
Fat (energy %)	22.9^a^	18.3, 27.7	29.9^b^	25.8, 34.5	29.1^b^	24.3, 34.0
Protein (g)	47.7^a^	36.1, 62.4	59.3^b^	45.3, 76.6	58.8^b^	42.8, 74.9
Fiber (g)	26.2^a^	18.7, 34.1	19.1^b^	14.4, 25.7	23.3^c^	17.0, 31.3
Sugar (g)	56.8^a^	38.1, 82.9	108.4^b^	78.2, 164.5	89.4^c^	64.3, 122.0
Saturated fat (g)	12.5^a^	8.4, 18.8	23.4^b^	16.9, 31.9	19.1^c^	13.2, 26.7
Saturated fat (energy %)	7.3^a^	5.3, 9.4	11.1^b^	9.0, 13.2	10.7^b^	8.4, 12.8
Polyinsatured fat (g)	11.0^a^	7.5, 15.1	14.2^b^	10.5, 20.4	10.8^a^	7.2, 15.8
Polyinsatured fat (energy %)	6.2^a^	5.0, 8.1	6.9^b^	5.3, 8.8	5.9^a^	4.7, 7.4
Cholesterol (mg)	146.5^a^	76.0, 246.3	227.4^b^	148.6, 341.7	206.1^b^	126.4, 291.7

*Values within a column with unlike superscript letter were significantly different (P < 0.016, using quantile regression model)*.

*^1^Expanded sample = 49, 575,450 adults*.

According to the healthy diet indicator components, adherence for fruits and vegetables was higher in the Diverse pattern, also in both sexes. The total HDI score was higher (*P* < 0.001) in the Rural pattern in men (6.1, 95% CI 5.9–6.2) and women (5.7, 95% CI 5.5–5.9) and the Diverse pattern (5.2, 95% CI 5.0–5.5 in men and 4.9, 95% CI 4.7–5.1 in women) P < 0.05, compared to the Westernized pattern (4.7, 95% CI 4.6–4.9 in men and 4.3, 95% CI 4.2–4.5 in women) (*P* < 0.05; Table 4).

**Table 4:** Adherence to the healthy diet indicator components and score by dietary pattern and gender, in Mexican adults.

	**Dietary patterns in men**					
	**Rural**		**Westernized**		**Diverse**	
**Men**	**%**	**95% CI**	**%**	**95% CI**	**%**	**95% CI**
Saturated fat, <10% of TE	88.0^a^	84.0–91.0	47.7^b^	42.2–53.3	37.9^b^	31.0–45.3
Polyinsaturated fat, 6–10% of TE	44.0	38.5–49.8	44.1	39.1–49.3	43.9	36.0–52.1
Protein, 10–15% of TE	85.2^a^	81.5–88.3	69.8^b^	65.1–74.2	52.4^c^	44.6–60.0
Carbohydrates, 50–70% of TE	66.3^a^	60.6–71.6	76.5^b^	72.2–80.3	72.3^ab^	64.5–79.0
Fiber, >25 g/d	79.2^a^	74.1–83.5	56.1^b^	50.3–61.6	71.8^a^	64.5–78.2
Fruits and vegetables, >400 g/d	20.0^a^	15.6–25.5	26.4^a^	21.8–31.6	64.8^b^	57.6–71.4
Legums and seeds, >30 g/d	52.2^a^	45.2–59.1	36.8^b^	31.9–42.0	42.0^ab^	34.3–50.1
Sugar, <10% of TE	94.0^a^	91.1–96.1	71.9^b^	66.5–76.7	87.7^a^	81.7–91.9
Cholesterol, <300 mg/d	76.9^a^	71.2–81.8	44.0^b^	39.0–49.2	49.2^b^	41.6–56.8
Total score HDI^†^	6.1^a^	5.9–6.2	4.7^b^	4.6–4.9	5.2^c^	5.0–5.5
**Women**						
Saturated fat, <10% of TE	78.4^a^	73.2–82.8	35.1^b^	30.1–39.7	37.4^b^	32.8–42.1
Polyinsaturated fat, 6–10% of TE	41.6	37.2–46.2	46.8	42.1–51.6	40.2	35.1–45.4
Protein, 10–15% of TE	80.6^a^	76.5–84.1	71.5^b^	67.1–75.5	58.1^c^	53.0–63.1
Carbohydrates, 50–70% of TE	70.4	65.2–75.1	74.1	69.6–78.1	69.4	63.4–74.8
Fiber, >25 g/d	55.3^a^	49.8–60.7	27.6^b^	23.3–32.3	43.8^a^	38.7–49.0
Fruits and vegetables, >400 g/d	13.3^a^	10.6–16.5	19.1^a^	15.5–23.2	55.7^b^	50.7–60.6
Legums and seeds, >30 g/d	50.6^a^	45.8–55.4	23.1^b^	19.3–27.5	28.1^b^	24.3–32.2
Sugar, <10% of TE	95.6^a^	94.0–96.7	72.3^b^	67.3–76.7	82.8^c^	78.8–86.2
Cholesterol, <300 mg/d	84.2^a^	78.9–88.4	66.2^b^	61.0–71.1	74.9^ab^	70.0–79.3
Total score HDI^†^	5.7^a^	5.5–5.9	4.3^b^	4.2–4.5	4.9^c^	4.7–5.1

*TE, Total Energy; HDI, Healthy Diet Indicator. †Values are means*.

*Percentages within a column with unlike superscript letters were significantly different (P < 0.016, using chi square test for categorical variables and T-test for HDI)*.

Table 5 shows the association analysis between dietary pattern and overweight and obesity and abdominal obesity, including interaction term of dietary pattern and gender. Using the Rural pattern as a reference, in men, we found a significant association between a Westernized pattern in (PR = 1.11, 95% CI 0.97–1.27), and Diverse Pattern (PR = 1.18, 95% CI 1.00–1.38), with overweight and obesity (*p* < 0.10). Including physical activity variable in the model, the association of Westernized and Diverse patterns was slightly higher (*p* < 0.05).

**Table 5:** Association between dietary patterns with overweight and obesity and abdominal obesity in Mexican adults, in the complete sample and sample with physical activity ENSANUT-2016^†^.

	**Complete sample**		**Sample with physical activity information^**†**^^†^**	
**Overweight and obesity^**‡**^**	**PR**	**CI, 95%**	**PR**	**CI, 95%**
Dietary pattern				
Rural	Reference		Reference	
Westernized	1.04	0.96–1.12	1.02	0.95–1.10
Diverse	0.96	0.89–1.04	0.95	0.88–1.03
Gender	0.84	0.75–94.3	0.83	0.74–0.92
Interaction (Westernized pattern—male gender)	1.11	0.97–1.27	1.14*	0.99–1.3
Interaction (Diverse pattern—male gender)	1.18*	1.00–1.38	1.20	1.02–1.41
**Abdominal obesity** ^**§**^				
Dietary pattern				
Rural	Reference		Reference	
Westernized	1.02	0.96–1.08	1.00	0.95–1.06
Diverse	0.97	0.93–1.02	0.96	0.92–1.01
Gender	0.64	0.56–0.73	0.63	0.55–0.72
Interaction (Westernized pattern—male gender)	1.15*	1.00–1.33	1.17*	1.00–1.36
Interaction (Diverse pattern—male gender)	1.27*	1.07–1.50	1.28*	1.07–1.52

*Poisson regression models adjusted by age, area, and region*.

*P-value for interaction term < 0.10.

*^‡^n = 5,735 adultos (expanded population = 49,575,450 adults)*.

*^§^n = 5,701 adultos (expanded population = 49,392,668 adults)*.

*††Physical activity was not available in 142 participants*.

Compared with the Rural pattern, Westernized, and Diverse patterns were associated with abdominal obesity in men (Westernized pattern PR = 1.15, 95% CI 1.00–1.33; Diverse pattern PR = 1.27, 95% CI 1.07–1.50, *p* < 0.05). In the sensitivity analysis, including physical activity variable the association was higher (*p* < 0.05).

## Discussion

This study shows that in Mexican adults, dietary patterns characterized by high energy, saturated fat, protein, and sugar intake and low fiber intake were associated with overweight and obesity (measured by BMI) and abdominal obesity in men, and these patterns have a lower diet quality indicator score. However, this association was not present in women.

We identified three dietary patterns: Rural, Diverse, and Westernized. These patterns are consistent with several studies carried out in adult populations. The Westernized pattern, in some studies called “modern” or “unhealthy,” is consistently energy dense, high in sugar and fats (total and saturated), and has been associated with overweight and obesity and abdominal obesity in adult populations (11, 17, 37, 38); these characteristics may explain the mechanism underlying this association.

The Rural pattern, as defined in our study, has been described in other studies as a “traditional pattern,” (11, 16, 19). The Rural pattern in men was protective against overweight and obesity, which is consistent with the results from a Brazilian study, in which a traditional Brazilian pattern (consisting of traditional foods like rice and beans) was protective against excess weight among men (11). In addition, a Mexican study found that adults that consumed the traditional pattern (with maize, maize foods, beans, and legumes) had a lower risk of presenting excess weight (19).

There are some hypotheses as to why the Rural pattern has a protective effect: (1) the high variety of healthy foods included in this pattern, (2) the high content of tortilla and legumes, and (3) the lower consumption of fat and sugar (39).

On the other hand, the results of the association between Westernized and Diverse patterns with obesity, have an unclear mechanism, but some hypotheses have been made regarding the role of the carbohydrates intake and energy density in appetite control. First, unlike the Rural pattern, in our study, Westernized y Diverse pattern were high in sugar. Westernized pattern was characterized for higher consumption of bread, salty snacks and potato. As we all know, refined grains are major source of dietary carbohydrate, and previous evidence indicates that high carbohydrate from refined grains are associated with obesity (40). Second, Diverse pattern was characterize by higher consumption of meat, ready to cereals, and dairy sweetened and non-sweetened beverages. Meat could increases the energy density, which may be a key component in body-weight regulation because it may alter appetite control signals (i.e., hunger and satiety). Although protein intake has been shown to increase satiety in intervention studies, the long-term effect of the consumption of a large amount of meat, remains unknown (41).

We did not find an association between dietary pattern and obesity in women. The available evidence of the relationship between dietary patterns and obesity stratified by sex has not been conclusive. A study carried out in the United States among Hispanic and non-Hispanic women found that a Westernized pattern was associated with a higher prevalence of overweight and obesity in both Hispanic and non-Hispanic white women (42). Another study carried out in Australian adults estimated the association between diet quality and change in obesity, finding an inverse association between diet quality and obesity in men, but not in women (43). However, there is a study than evidence that the main difference is that men habits were characterized by travel and eating out much more, consume more alcohol and tobacco, which represent more energy compared to women (44). The lack of association in women may be due to three reasons: (1) possible underreporting of energy in those with a higher BMI (women presented more overweight and obesity prevalence) (45), (2) the possibility that overweight women adopt a healthier diet to manage their weight (Dieting is an issue mostly observed in female participants) (46), and (3) the possibility of differences in food groups distribution even in the same dietary pattern.

Regarding the characteristics of the dietary patterns and obesity in adults, the findings obtained here are consistent with those found in a longitudinal study of adults in the United States. It demonstrated that individuals who followed a healthy dietary pattern had lower BMI, waist circumference, and blood pressure compared to more eastern dietary patterns. Individuals who followed a dietary pattern characterized by red meat, potatoes, and sweet foods had higher levels of glycosylated hemoglobin (47).

Furthermore, our results showed higher adherence to the HDI components in the Rural pattern than in the Westernized pattern, in both genders. The analysis with this indicator confirms the results with cluster analysis, showing better diet quality in the rural pattern compared to the Westernized pattern.

### Limitations of the Study

This study has some limitations. First, as the diet information is self-reported, the recall bias cannot be eliminated. However, the interviewers were trained in the FFQ methodology, which could have minimized it. Second, the *a posteriori* approach to derive dietary pattern includes certain subjectivity, such as the selection of food groups and number of patterns (48). However, once the dietary patterns were identified, we evaluated their adherence to diet quality recommendations with an *a priori* index, and we found that the results with both methodologies were consistent. The third possible limitation is the relatively short period for which dietary information was collected (7-day FFQ), which may not have been enough time to capture the intake variability in women. This could explain why we did not find an association between diet and overweight and obesity in women. Another limitation is that we did not count with a precise measurement of physical activity such as accelerometry, however, we got information from self-report of physical activities in a part of the sample, which was useful to adjust the final model, founding higher associations between Westernized and Diverse patterns and obesity in male population.

### Strengths of the Study

This study has some strengths as well. Data come from a nationally representative nutrition survey. The FFQ that was used has been validated to identify dietary patterns in the Mexican adult population (49). In addition, trained personnel obtained standardized anthropometrical measures to avoid a systematic error. This despite the fact that in recent years, strategies to improve nutrition have been implemented in Mexico, such as the tax on sugar-sweetened beverages and non-essential energy-dense foods (50). Also, both cluster and diet quality methodologies used to identify dietary patterns support the evidence that a healthier diet is important for preventing obesity in the adult population. However, it is necessary to analyze other factors to understand how dietary patterns are related to obesity in women.

The findings of the present study confirm the impact of dietary patterns on the weight of Mexican adults and expand the overview to investigate behavioral aspects in men that could relate biological aspects. The food groups and moreover dietary patterns highlight the importance of epigenetic studies focused on obesity development.

In conclusion, Westernized and Diverse patterns characterized by sweetened non-dairy beverages, fast food, bakery and cookies, corn based salty dishes, bread, candies, and salty snacks are associated with overweight and obesity and abdominal obesity in Mexican men. Our findings are politically relevant, and align with evidence that highly energy dense and ultra-processed foods are associated with obesity. They also emphasize the urgent need for future studies which should examine dietary patterns with a gender perspective, as social roles could be important in the result of strategies and recommendations to promote healthy diets. Dietary pattern research has great potential for use in nutrition policy, particularly as it demonstrates the importance of total dietary intake in health promotion.

## Data Availability Statement

The raw data supporting the conclusions of this article will be made available by the authors, without undue reservation.

## Ethics Statement

The studies involving human participants were reviewed and approved by National Institute of Public Health in Mexico (INSP). The patients/participants provided their written informed consent to participate in this study.

## Author Contributions

SR-R is responsible for conceiving the study, developing the overall research plan, and overseeing the study. SR-R and BM-T analyzed the data and primarily responsible for the final content. SR-R wrote the first draft and BM-T and DG-C added important intellectual content. LC-N and TS-L did the final revision and provided contributions. All authors read and approved the final submitted manuscript.

## Funding

This project was partially funded by Bloomberg Philanthropies.

## Conflict of Interest

The authors declare that the research was conducted in the absence of any commercial or financial relationships that could be construed as a potential conflict of interest.

## Publisher's Note

All claims expressed in this article are solely those of the authors and do not necessarily represent those of their affiliated organizations, or those of the publisher, the editors and the reviewers. Any product that may be evaluated in this article, or claim that may be made by its manufacturer, is not guaranteed or endorsed by the publisher.

## Footnotes

^1^Instituto Nacional de Salud Publica. Compilación de bases de datos de valor nutritivo de los alimentos. [National Institute of Public Health. Food Composition Table]. Cuernavaca: National. Institute of Public Health (Unpublished).
