# Supplementary material for: Westernized and Diverse Dietary Patterns Are Associated With Overweight-Obesity and Abdominal Obesity in Mexican Adult Men
Source: Front Nutr. 2022 Jun 24;9:891609. doi: 10.3389/fnut.2022.891609 (PMC9263742; doi:10.3389/fnut.2022.891609)
Supplement: Supplementary file 1 [file Table_1.docx]

**Table S1. Examples of food included in the food groups**

| Food group | Examples of foods included in the group |
| --- | --- |
| Dairy sweetened beverages | Atole with milk^a^, smoothies, flavored milk, and milk beverages with sugar |
| Dairy non sweetened beverages | Plain milk |
| Sweetened non-dairy beverages | Industrialized juice and soft drinks of any flavor, juice made from any natural fruit, sports drinks, and *Aguas Fresca*^b^ |
| Non sweetened, non-dairy beverages | Coffee or tea without sugar, diet soda, and *Agua Fresca*^b^ without sugar |
| Fruits | Any kind of fruit |
| Vegetables | Any kind of vegetables |
| Dairy products no beverages | Cheeses of any kind, and solid yogurt |
| Legumes | Beans, chickpeas, and lentil |
| Cereal based salty dishes | Rice of pasta of any preparation |
| Corn based salty dishes | Mexican food as tacos, sopes, and quesadillas |
| Fast food | Hamburger, hot dog, and pizza |
| Egg | Egg of any species |
| Meat and poultry | Pork, beef, chicken and any kind of meat |
| Processed meat | Sausages and ham |
| Bakery and cookies | Pastries and cokies |
| Candies | Candies |
| Desserts | Desserts, ice cream, and cakes |
| Salty snacks | Chips, popcorn, and potato sticks |
| Seeds | Peanut, and nuts |
| Added fats | Oils, margarine, and milk cream |
| Tortilla | Corn tortilla |
| Soup | Cream soups based on milk, soup with pasta or vegetables |
| Ready to eat cereals | Breakfast cereal based in oat, rice, wheat or other grain (Chococrispis, Zucaritas, Nesquik, and Zucoso) |
| Bread | Oatmeal, whole-grain cereal, salty bread, and non-sweet whole-grain cereals |
| Potatoes | Potato of any preparation |
| ^a^ Beverage made with wheat, oat or maize flour, milk and sugar | |
| ^b^ Beverage made with water, fruit and sugar | |
